# Recent advances in use of bio-inspired jellyfish search algorithm for solving optimization problems

**DOI:** 10.1038/s41598-022-23121-z

**Published:** 2022-11-10

**Authors:** Jui-Sheng Chou, Asmare Molla

**Affiliations:** National Taiwan University of Science and Technology, Taipei, Taiwan

**Keywords:** Applied mathematics, Computational science, Civil engineering, Electrical and electronic engineering, Energy science and technology, Engineering, Mathematics and computing

## Abstract

The complexity of engineering optimization problems is increasing. Classical gradient-based optimization algorithms are a mathematical means of solving complex problems whose ability to do so is limited. Metaheuristics have become more popular than exact methods for solving optimization problems because of their simplicity and the robustness of the results that they yield. Recently, population-based bio-inspired algorithms have been demonstrated to perform favorably in solving a wide range of optimization problems. The jellyfish search optimizer (JSO) is one such bio-inspired metaheuristic algorithm, which is based on the food-finding behavior of jellyfish in the ocean. According to the literature, JSO outperforms many well-known meta-heuristics in a wide range of benchmark functions and real-world applications. JSO can also be used in conjunction with other artificial intelligence-related techniques. The success of JSO in solving diverse optimization problems motivates the present comprehensive discussion of the latest findings related to JSO. This paper reviews various issues associated with JSO, such as its inspiration, variants, and applications, and will provide the latest developments and research findings concerning JSO. The systematic review contributes to the development of modified versions and the hybridization of JSO to improve upon the original JSO and present variants, and will help researchers to develop superior metaheuristic optimization algorithms with recommendations of add-on intelligent agents.

## Introduction

Optimization is a process that is used to find the best inputs to maximize/minimize outputs at affordable computational cost^1, 2^. The complexity of engineering optimization problems is increasing. Classical gradient-based optimization algorithms have a limited ability to solve complex optimization problems using conventional mathematical methods^3–5^. Obviously, some traditional methods can be used to solve optimization problems, but they may not yield optimal results. Furthermore, traditional methods cannot resolve all difficult non-linear problems in an acceptable time^6, 7^. Metaheuristics have become more popular than exact methods for solving optimization problems because of their simplicity and the robustness of the results that they yield^8–10^. Population-based bio-inspired algorithms have recently been demonstrated to perform well in solving a wide range of optimization problems.

Recently developed original algorithms include forensic-based investigation algorithm (FBI)^8^, the slime mould algorithm (SMA)^11^, the group teaching optimization algorithm (GTOA)^12^, dynamic group optimization (DGO)^13^, the African vultures optimization algorithm (AVOA)^14^, the Rao-3 algorithm^15^, the gorilla troops optimizer (GTO)^16^, smell agent optimization (SAO)^17^, the sparrow search algorithm (SSA)^18^, the artificial ecosystem optimizer (AEO)^19^, the starling murmuration optimizer (SMO)^20^, the dwarf mongoose optimization algorithm (DMOA)^21^, the war strategy optimization algorithm (WSOA)^22^, the dynamic butterfly optimization algorithm (DBOA)^23^, the artificial hummingbird optimization technique (AHOT)^24^, and the antlion optimization algorithm (ALOA)^25^.

Newly enhanced algorithms include the fractional-order modified Harris hawks optimizer (FMHHO)^26^, the modified manta ray foraging optimization algorithm (MMRFOA)^27^, an enhanced slime mould algorithm^28^, the hybrid marine predator algorithm (HMPA)^29^, partitioned step particle swarm optimization (PSPSO)^30^, the improved chimp optimization algorithm (ICHOA)^31^, the high performance cuckoo search algorithm (HPCSA)^32^, the comprehensive learning marine predator algorithm (CLMPA)^33^, the enhanced sparrow search algorithm (ESSA)^34^, the hybrid algorithm that is known as three-learning strategy PSO (TLS-PSO)^35^, the enhanced shuffled shepherd optimization algorithm (ESSOA)^36^, the hybrid salp swarm algorithm with teaching-learning-based optimization (HSSATLBO)^37^, and an enhanced hybrid of crisscross optimization and the arithmetic optimization algorithm (CSOAOA)^38^. Both original and enhanced metaheuristic optimization algorithms are used in a wide range of fields, including engineering, business, transportation, energy, and even the social sciences.

Bio-inspired metaheuristics can be classified into four main categories based on the source of inspiration for their development; these are evolution-based, physics-based, swarm-based, and human-based. Recently, a novel swarm-based metaheuristic algorithm, called the jellyfish search optimizer (JSO)^39^, was developed based on the food-finding behavior of jellyfish in the ocean. According to Chou and Truong^39^, jellyfish move in the ocean in search of planktonic organisms such as fish eggs, larvae, and phytoplankton, and their movement patterns are of two major types, which are (1) following the ocean current and (2) within a jellyfish swarm. The simulation of JSO involves two phases which are diversification and intensification of the search. A time control mechanism governs the switching between these patterns of movement.

JSO can be used in conjunction with other AI-related methods, such as machine and deep learning tools, to optimize their hyper-parameters. The success of JSO in solving a wide range of optimization problems has inspired the present authors to discuss comprehensively the latest findings concerning JSO. This paper reviews various issues around JSO, including its inspiration, variants, and applications, and will provide the latest developments and research findings associated with JSO. The rest of the paper is organized as follows. “Bio-inspired jellyfish search algorithm” outlines the theoretical framework of JSO and explains its implementation in detail. “Recent improvements on and variants of JSO” presents improvements to JSO and corresponding, recently developed variants. “Applications” briefly explains novel applications of JSO in the real world and the field of artificial intelligence. “Discussion” compares performance among, and discusses potential enhancements of, JSO variants. “Conclusion and recommendations” draws conclusions and offers recommendations.

## Bio-inspired jellyfish search algorithm

The jellyfish search optimizer (JSO) is a newly developed swarm-based optimization algorithm that was designed and proposed by Chou and Truong^39^. The following sections discuss the inspiration by jellyfish behavior and mathematical formulations of population initialization, the movement of jellyfish in an ocean current and in a swarm, time control mechanism, and the boundary conditions. The advantages and limitations of the algorithm will also be considered.

### Inspiration by jellyfish behavior

Jellyfish live in water of various depths and temperatures around the world. They are shaped like bells; some have a diameter of less than a centimeter while others are very large^39, 40^. They have a wide range of colors, sizes, and shapes. All of the many species exhibit particular adaptations to the oceanic environment. Their methods of feeding vary: some jellyfish use tentacles to bring food to their mouths while others use filter-feeding to eat whatever the current brings them; yet others actively hunt prey and immobilize them by stinging them with their tentacles^39, 41^. Jellyfish use their tentacles to sting their prey, and then release a venom that paralyzes it. They do not attack creatures, but those who swim up against or touch them may be stung to death. Jellyfish are most dangerous when gathered together in a jellyfish bloom^39, 42^.

Jellyfish have special features that enable them to control their movements. Their undersides close like an umbrella, pushing water out to propel their bodies forward. Despite this ability, they mostly drift in the water, so their motion is determined by currents and tides^43^. Jellyfish can form a swarm, and a large mass thereof is called a jellyfish bloom^39, 44^. In particular, jellyfish are weak swimming organisms and their orientations with respect to currents are key to the maintenance of blooms and ensuring that they do not become stranded. Numerous factors govern the formation of a swarm, including ocean currents, available nutrients, the availability of oxygen, the presence of predators, and temperature. Among these factors, ocean currents are the most important as they can collect jellyfish into a swarm^39, 45^.

**Figure 1:**
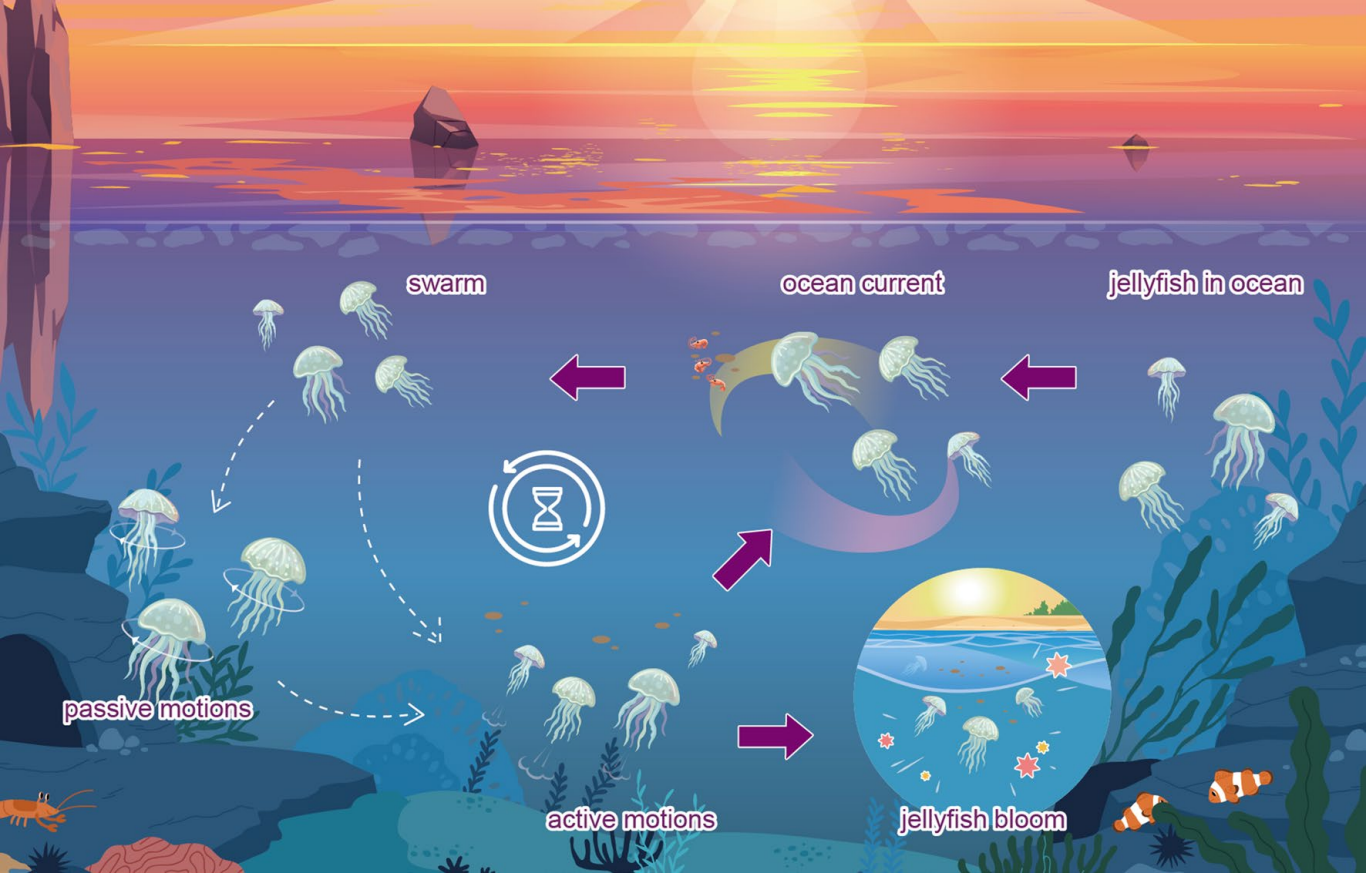
Search behavior and movement of jellyfish in ocean.

The swarming of jellyfish, their movements inside swarms and the formation of blooms as jellyfish follow ocean currents have distributed jellyfish species almost everywhere in the ocean. The quantity of food at sites that are visited by a jellyfish varies. Jellyfish thus compare the amounts of food available at various sites and identify the best of them. Accordingly, a new algorithm, called the jellyfish search optimizer (JSO)^39^ and inspired by the search behavior and movements of jellyfish in the ocean is developed. Figure 1 shows the simulated search behavior and movement of jellyfish in ocean^46, 47^. In the next section, the behavior and movements of jellyfish in the ocean will be mathematically modeled, and a bio-inspired optimization algorithm that is based on the mathematical model will then be presented^39^.

### Schematic representation

**Figure 2:**
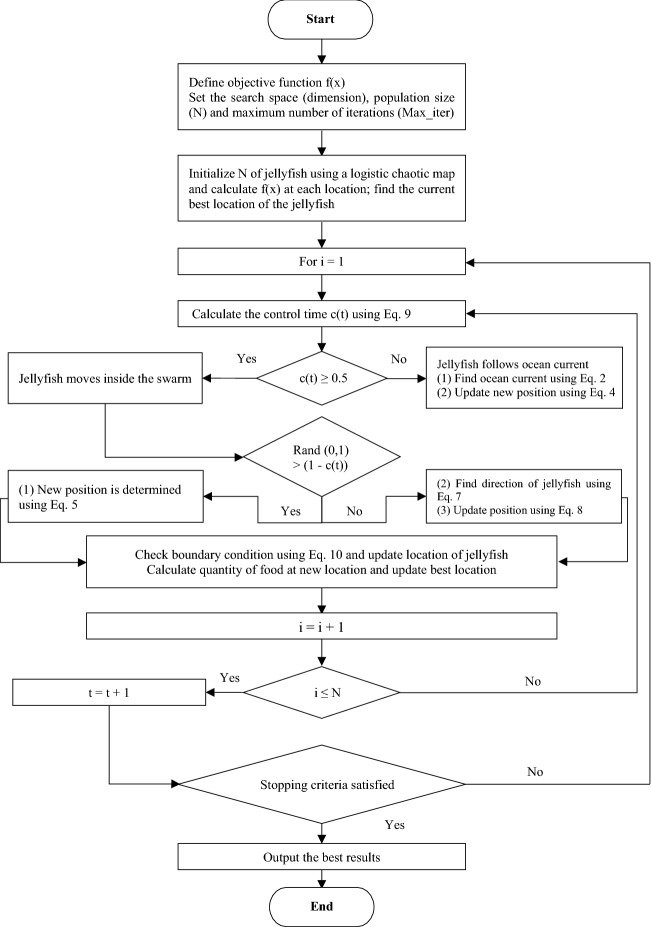
Schematic flowchart of SOJS algorithm, adapted from Ref.^39^.

**Figure 3:**
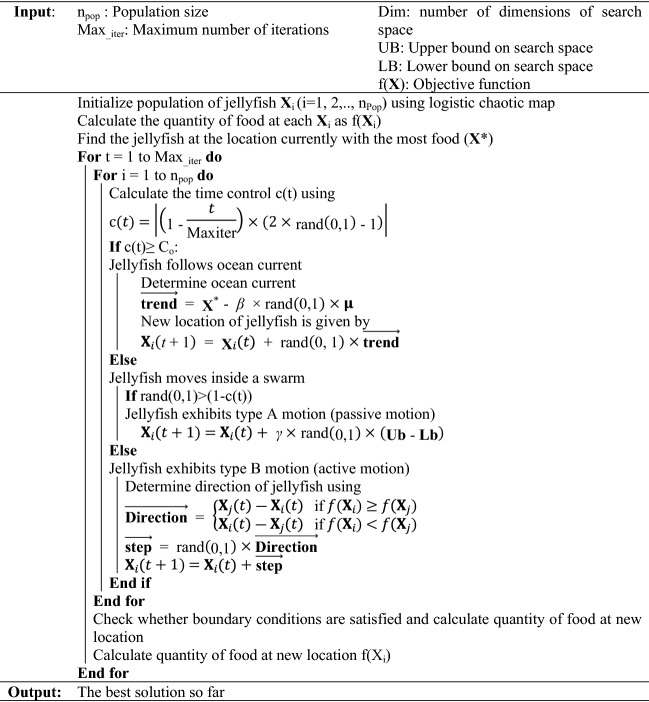
Pseudocode of JSO algorithm, adapted from Ref.^39^.

The two main phases of a metaheuristic optimization algorithm are exploration and exploitation. In JSO, movement toward an ocean current is exploration, movement within a jellyfish swarm is exploitation, and a time control mechanism switches between them. Initially, the probability of exploration exceeds that of exploitation to find areas that contain promising optimal positions with respect to finding of food; over time, the probability of exploitation becomes much higher than that of exploration, and the jellyfish identify the best location inside the searched areas. Figures 2 and 3 present the flowchart and pseudocode of the single objective jellyfish search (SOJS) optimizer, respectively.

### Mathematical formulation

The proposed optimization algorithm is based on three idealized rules^39^.

- Jellyfish either follow the ocean current or move inside the swarm; a time control mechanism governs the switching between these types of movement.
- Jellyfish move in the ocean in search of food. They are more attracted to locations where more food is present.
- The quantity of food found is determined by the location and the corresponding objective function.

#### Population initialization

The population of an artificial optimization algorithm is normally initialized at random. The disadvantages of this method are its slow convergence and its tendency to become trapped at local optima as a result of low population diversity. To improve the diversity of the initial population while maintaining simplicity, JSO uses a chaotic map^39^ known as the logistic map^48^ which provides more diverse initial populations than does random selection and it yields a lower probability of premature convergence as shown in Eq. (1)^39^.

1 $${\text{X}}_{i+1}= \eta{\text{X}}_{i}\left(\text{1 }- \, {\text{X}}_{i}\right)\text{, 0 }\le {\mathbf{X}}_{i}\le 1,$$

where, ${\text{X}}_{i+1}$ is the location of the *i*th jellyfish;${\mathbf{X}}_{i}$ is the logistic chaotic value of the location of the *i*th jellyfish; ${\mathbf{X}}_{i}$ is used to generate an initial population of jellyfish; ${\mathbf{X}}_{o}\in \left(\text{0,1}\right)\text{,}{ \, {\text{X}}}_{o}\notin \left\{\text{0.0,0.25,0.75,0.5,1.0}\right\},$ and the parameter $\eta$ is set to 4.0^39, 49, 50^.

#### Following ocean current

An ocean current contains large quantities of nutrients, so the jellyfish are attracted to it. The direction of the ocean current ($\overrightarrow{\text{trend}}$) is determined by averaging all of the vectors from all jellyfish in the population to the jellyfish that is currently in the best location. The ocean current can be simulated using Eq. (2) ^39^.

2 $$\overrightarrow{\text{trend}} = {\text{X}}^{*}- \, {\upbeta }{\times }rand\left(\text{0,1}\right)\times {\varvec{\upmu}},$$

where ${\mathrm{X}}^{*}$ is the jellyfish that is currently is in the best location in the swarm; ${\varvec{\upmu}}$ is the mean location of all jellyfish, and $\beta$ > 0 is a distribution coefficient that is related to the length of the ocean current ($\overrightarrow{\text{trend}}$). Based on the results of a sensitivity analysis in numerical experiments^39^, $\beta$ =3. Thus, the new location of each jellyfish is given by Eqs. (3) and (4)^39^.

3 $${\mathbf{X}}_{i}\left({\text{t}}+ \text{1} \right)\text{ = }{\text{X}}_{i}\left(t\right)\text{ + }rand\left(\text{0, 1}\right)\times \overrightarrow{\text{trend}},$$

4 $${\mathbf{X}}_{i}\left({\text{t}}+ \text{1} \right) = {\text{X}}_{i}\left(t\right) + rand\left(\text{0, 1}\right)\times {\mathbf{X}}^{*}\text{- }{\upbeta }\times \text{ rand}\left(\text{0,1}\right)\times\upmu ,$$

where ${\mathbf{X}}_{i}(t)$ is the current location of the jellyfish and ${\mathbf{X}}_{i}({\text{t}}+ \text{1} )$ is the new location of the jellyfish.

#### Jellyfish swarm

In a swarm, jellyfish exhibit passive (type A) and active (type B) motions^42, 51^. Initially, when a swarm has just been formed, most jellyfish exhibit type A motion. Over time, they increasingly exhibit type B motion.

Type A motion is that of jellyfish around their own locations. The corresponding updated location of each jellyfish is given by Eq. (5)^39^.

5 $${\mathbf{X}}_{i}\left(t+1\right)={\mathbf{X}}_{i}\left(t\right)+ \, {\upgamma}\times rand\left(\text{0,1}\right)\times \left({\textbf{Ub}}-{\textbf{Lb}}\right),$$

where $\mathbf{U}\mathbf{b}$ and $\mathbf{L}\mathbf{b}$ are the upper bound and lower bound on the search space, respectively, and $\gamma$ > 0 is a motion coefficient, which is related to the length of the motion around each jellyfish’s location. The results of a sensitivity analysis in a numerical experiment^39^ yield $\gamma$ = 0.1.

To simulate type B motion, a jellyfish (*j*) other than the one of interest is selected at random and a vector from the jellyfish of interest (*i*) to the selected jellyfish (*j*) is used to determine the direction of movement. When the quantity of food at the location of the selected jellyfish (*j*) exceeds that at the location of the jellyfish (*i*) of interest, the latter moves toward the former; it moves directly away from it if the quantity of food available to the selected jellyfish (*j*) is lower than that available to the jellyfish of interest (*i*). Consequently, each jellyfish in a swarm moves toward a better location to find food. Equations (6), (7) and (8) simulate the direction of motion and the updated location of a jellyfish^39^, respectively. This movement is considered to be effective exploitation of the local search space.

6 $$\overrightarrow{\text{Step}}\text{ = }rand\left(\text{0,1}\right)\times \overrightarrow{\text{Direction}},$$

7 $$\overrightarrow{\text{Direction}}\text{ = }\left\{\begin{array}{c}{\mathbf{X}}_{j}(t)-{\mathbf{X}}_{i}(t) \, \, \, {\text{i}}{\text{f}} \, f({\mathbf{X}}_{i})\ge f({\mathbf{X}}_{j})\\ {\mathbf{X}}_{i}\left(t\right)-{\mathbf{X}}_{j}\left(t\right) \, \, \, {\text{i}}{\text{f}} \, f\left({\mathbf{X}}_{i}\right)<f\left({\mathbf{X}}_{j}\right),\end{array}\right.$$

8 $$\mathrm{Hence}, {\mathbf{X}}_{i}\left(t+1\right)={\mathbf{X}}_{i}\left(t\right)+\overrightarrow{\text{Step}},$$

where $f$ is an objective function of location X.

#### Time control mechanism

The ocean current contains large amounts of nutritious food so jellyfish are attracted to it^43^. Over time, more jellyfish gather together and a swarm is formed. When the temperature or wind changes the ocean current, the jellyfish in the swarm move toward another ocean current, and another jellyfish swarm is formed. The movements of jellyfish inside a jellyfish swarm are type A (passive motions) and type B (active motions), between which the jellyfish switch. Type A is favored in the beginning; as time goes by, type B becomes more favored^39^.

The time control mechanism is introduced to simulate this situation. To regulate the movement of jellyfish between following the ocean current and moving inside the jellyfish swarm, the time control mechanism includes a time control function $\mathrm{c}\left(t\right)$ and a constant ${\mathrm{c}}_{o}$. The time control function is a random value that fluctuates from zero to unity over time. Equation (9)^39^ provides the time control function over time; when its value exceeds ${\mathrm{c}}_{o}$, the jellyfish follow the ocean current. When its value is less than ${\mathrm{c}}_{o}$, they move inside the swarm. An exact ${\mathrm{c}}_{o}$ value is not known and the time control varies randomly from zero to one. Hence, ${\mathrm{c}}_{o}$ is set to 0.5, which is the mean of zero and one.

9 $$\mathrm{c}\left(t\right)=\left|\left(\text{1 - }\frac{t}{\text{Maxiter}}\right)\times \left(2\times rand\left(\text{0,1}\right) \text{- 1}\right)\right|,$$

where $\mathrm{c}\left(t\right)$ is the time control function;${\mathrm{c}}_{o}$ is a constant that is set to 0.5;$t$ is the time that is specified by the iteration number, and ${\text{Maxiter}}$ denotes the maximum number of iterations, which is an initialized parameter.

The term $\left(\text{1-c}\left(t\right)\right)$ is used to simulate the movement inside a swarm (type A or B)^39^. When $rand\left(\text{0,1}\right)$ exceeds $\left(\text{1-c}\left(t\right)\right)$, the jellyfish exhibits type A motion. When $rand\left(\text{0,1}\right)$ is lower than $\left(\text{1-c}\left(t\right)\right)$, the jellyfish exhibits type B motion. Since $\left(\text{1-c}\left(t\right)\right)$ increases from zero to one over time, the probability that $rand\left(\text{0,1}\right)>\left(\text{1-c}\left(t\right)\right)$ initially exceeds the probability that $rand\left(\text{0,1}\right)<\left(\text{1-c}\left(t\right)\right)$. Therefore, type A motion is preferred to type B. As time goes by, $\left(\text{1-c}\left(t\right)\right)$ approaches one, and the probability that $rand\left(\text{0,1}\right)<\left(\text{1-c}\left(t\right)\right)$ ultimately exceeds the probability that $rand\left(\text{0,1}\right)>\left(\text{1-c}\left(t\right)\right)$. So, type B motion is favored.

#### Boundary conditions

Oceans are located around the world. The earth is approximately spherical, so when a jellyfish moves outside the bounded search area, it will return to the opposite bound Eq. (10) presents this re-entering process^39^.

10 $${\text{X}}_{i\text{,d}}^{^{\prime}}\text{ = }\left\{\begin{array}{c}\left({\text{X}}_{i\text{,d}} \, - \, {\text{U}}_{\text{b,d}}\right) \, \text{+} \, {\text{L}}_{\text{b,d}} \, \, \, {\text{i}}{\text{f}} \, {\text{X}}_{i\text{,d }}\text{>} \, {\text{U}}_{\text{b,d}}\\ \left({\text{X}}_{i\text{,d}} \, - \, {\text{L}}_{\text{b,d}}\right) \, \text{+} \, {\text{U}}_{\text{b,d}} \, \, \, {\text{i}}{\text{f}} \, {\text{X}}_{i\text{,d}} \, \text{<} \, {\text{L}}_{\text{b,d}},\end{array}\right.$$

where,${\text{X}}_{i\text{,d}}$ is the location of the *i*th jellyfish in the dth dimension;${\text{X}}_{i\text{,d}}^{^{\prime}}$ is the updated location after the boundary constraints have been applied, and ${\text{U}}_{\text{b,d}}$ and ${\text{L}}_{\text{b,d}}$ are the upper bound and lower bound in the dth dimension of the search space, respectively.

#### Advantages and limitations

The jellyfish search optimizer (JSO) is one of the newest swarm intelligence algorithms; it is inspired by the behavior of jellyfish in the ocean. JSO converges more rapidly and has a stronger search ability than classic optimization methods with few algorithmic parameters. Furthermore, JSO maintains a better balance between exploration and exploitation than other algorithms. Maintaining a balance between the exploration and exploitation of a search space greatly influences the performance of an algorithm. Exploration refers to visiting entirely new regions within the search space. Exploitation refers to visiting those regions of a search space within the neighborhood of previously visited points. JSO can also be used in conjunction with other artificial intelligence (AI)-related techniques.

JSO has been shown to be efficient in solving numerous standard benchmark problems and to have both constrained and unconstrained real-world applications. However, JSO has some minor shortcomings. The algorithm may sometimes become stuck in local optima, suffer from premature convergence^52^, or take a long time to converge^55^. Therefore, it has room for improvement. Necessary improvements can be made by regulating the exploration and exploitation search, preserving the diversity of its search, and accelerating convergence. The following section introduces JSO variants that have been proposed in the literature with improved optimization performance.

## Recent improvements on and variants of JSO

**Figure 4:**
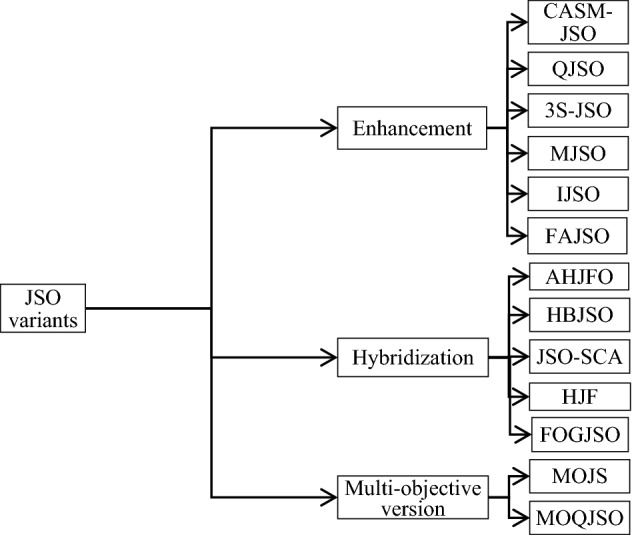
JSO enhancement and its variants.

The literature includes variants or enhancements of JSO, hybridization, and multi‑objective versions of JSO, as shown in Fig. 4. This section will present an overview of these variants of JSO.

### Intelligent agent modification in original JSO

Table 1 presents the enhancements of the JSO variants in recent studies with respect to population diversity, exploration, exploitation, boundary handling, and the time control mechanism. This subsection briefly describes the intelligent agents that have been proposed.

**Table 1:** Enhancements of JSO.

Authors	Algorithm	Modification/enhancement				
		Population diversity	Exploration	Exploitation	Boundary handling	Time control mechanism
Rajpurohit and Sharma^77^	CASM-JSO	v	–	–	–	–
Kaveh et al.^52^	QJSO	–	v	v	v	v
Bujok^53^	3S-JSO	v	v	–	–	–
Manita and Zermani^54^	MJSO	–	–	v	–	–
Abdel-Basset et al.^55^	IJSO	–	–	v	–	–
Truong and Chou^47^	FAJSO	–	v	v	–	v

#### Increasing population diversity

Bio-inspired optimization algorithms are sometimes enhanced to improve on one or more processes such as those associated with the algorithm parameters. Rajpurohit and Sharma^56^ proposed a chaotic active swarm method (CASM) for initializing the population in JSO. They tested seven chaotic maps (logistic map, circle map, Kent map, piecewise map, sine map, sinusoidal map and tent map) and compared them on benchmark functions and classical constrained engineering design problems. These chaotic maps are implemented to modify the active swarm motion step of the original JSO algorithm. The analysis of the results suggests that the sinusoidal map outperforms all the other chaotic maps that are adopted in JSO to increase population diversity.

#### Exploration boosting

In the early stages of the search process, the mean of the jellyfish swarm moves toward the location of the current best jellyfish of the swarm. This situation may lead to undesirable premature convergence in the early stages of the search because the swarm probably does not comprise high-quality jellyfish. To address this issue and to improve the exploration capacity of the original JSO, a quantum-based update rule named quantum JSO (QJSO) was proposed by Kaveh et al.^52^, based on quantum theory for use in the exploration phase of the classical JSO.

The QJSO involves the quantum-based update rule, which is applied to encourage diversification in the search space. The local attractor of each jellyfish of the swarm is determined using Eq. (11)^52^:

11 $${\mathbf{X}}_{i\text{,d}}^{t}\left(t\right)\text{ = }{\mathbf{X}}_{i\text{,d}}\left({\text{t}}\right)\times rand{1}_{i\text{,d}}\left(\text{0,1}\right)+\left(\text{1} - rand{1}_{i\text{,d}}\left(\text{0,1}\right)\right)\times {\mathbf{X}}_{\mathrm{d}}^{\text{best}},$$

where ${\mathbf{X}}_{i\text{,d}}^{t}(t)$ is the local attractor of the ith jellyfish of the swarm;${\mathbf{X}}_{i\text{,d}}({\text{t}})$ is the current location of the *i*th jellyfish in the swarm; ${\mathbf{X}}_{\mathrm{d}}^{\text{best}}$ is the best jellyfish identified so far, and *rand*1 is a random number that is uniformly distributed on (0, 1). Equation (11) indicates that ${\mathbf{X}}_{i\text{,d}}\left({\text{t}}\right),$ the local attractor of the *i*th jellyfish, lies on the line that connects ${\mathbf{X}}_{i\text{,d}}({\text{t}})$ and ${\mathbf{X}}_{\mathrm{d}}^{\text{best}}$ so that it moves following ${\mathbf{X}}_{i\text{,d}}({\text{t}})$ and ${\mathbf{X}}_{\mathrm{d}}^{\text{best}}.$

The location of each jellyfish is updated according to Eqs. (12) and (13)^52^.

12 $${\mathbf{X}}_{i\text{,d}}({\text{t}}+ \text{1} )\text{ = }{\mathbf{X}}_{i\text{,d}}^{t}(t)\text{ } + (\text{-1}{)}^{randi\left(\left[\text{1,2}\right]\right)}\times {\text{log}}\left(\frac{1}{rand{2}_{i\text{,d}}}\left(\text{0,1}\right)\times \beta \left(t\right)\right)\times \left|{\upmu }_{\mathrm{d}}-{\mathbf{X}}_{i\text{,d}}\left(t\right)\right|,$$

13 $$\mathrm{where } \ \beta \left(t\right)\text{ = 1-}\left(\text{1} - rand{3}\left(\text{0,1}\right)\right)\times \left(\frac{1}{\text{Maxiter}}\right).$$

${\mathbf{X}}_{i\text{,d}}({\text{t}}+ \text{1} )$ represents the new location; ${\mathbf{X}}_{i\text{,d}}^{t}(t)$ is the current location of the *i*th jellyfish; *rand*2 and *rand*3 are random numbers that are uniformly distributed in (0, 1); $randi$(1, 2) generates uniformly distributed random integers in (1, 2); ${\upmu}_{\mathrm{d}}$ is the mean location of all jellyfish of the swarm, and $\beta (t)$ controls the convergence of the jellyfish toward the best location that has been identified so far.

In the original JSO, distribution coefficient *β* was used in the global exploration phase of following the ocean current (Eq. (2)). Chou and Truong^39^ recommended setting *β* = 3, which achieves very promising results. The distribution coefficient *β* influences the degree of diversity of the population that is used to determine a new jellyfish position. A higher *β* means the new position is farther from the current best position. Naturally, when the diversity of the population is small, a higher *β* helps to generate new positions at a greater distance from the best solution. Bujok^53^ proposed an enhancement to this dynamic diversity control that uses a transformed adaptation of the distribution coefficient ($\beta$) during the search, based on the current diversity of the jellyfish population, which is estimated using Eq. (14)^53^:

14 $${D}_{i}=\sqrt{\frac{1}{N}{\sum }_{i= \text{1} }^{N}{\sum }_{j= \text{1} }^{D}{\left({\mathbf{X}}_{ij}{-\upmu}\right)}^{2}}{, \upmu}=\frac{1}{N}{\sum }_{i= \text{1} }^{N}{\mathbf{X}}_{ij},$$

where *D* represents the dimensionality of the problem; *N* is the population size; ${\mathbf{X}}_{ij}$ is the location of jellyfish (*i*) at dimension (*j*); and ${\upmu}$ is the mean location of all jellyfish of the swarm.

Bujok further used an eigen transformation-method to improve the diversity of population. The eigen-transformation in JSO is controlled by two parameters—the proportion of individuals that are chosen from the current population and used to calculate the covariance matrix and the pseudo-spectrum that is obtained using the eigenvector method. The former controls the proportion of the population *N* that is used to compute eigenvector ***B*** in Eq. (15)^53^ .

15 $$\mathbf{C= }{\varvec{B}}{\text{ D}}^{2}{{\varvec{B}}}^{T}$$

where ${\textbf{C}}$ is the covariance matrix of the selected part of the population; ${\varvec{B}}$ is the eigenvector and ${\text{D}}$ is the eigenvalue. Then, the eigenvectors are employed to transform the original coordinates of the selected individual.

In each JSO generation, either the original coordinate system or the new eigen-transformed coordinate system is applied, as determined by the second control parameter, the pseudo-spectrum, using the eigenvector method. If rand < pseig, then the eigen transformation is applied to the whole generation and several subsequent steps of JSO are affected by this transformation^53^. In the following ocean current phase (Eq. (4)), when the new position ${\mathbf{X}}_{i}({\text{t}}+ \text{1} )$ is computed, all three individual positions, ${\text{X}}_{i}(t)$, ${\mathbf{X}}^{*}$ and the average population vector, are transformed using the eigen-transformation that is given by Eq. (16)^53^. Similarly, in the active motion phase, the eigen-transformation is applied.

16 $${{\mathbf{X}}^{^{\prime}}}_{i}= {\varvec{B}}^{\mathrm{T}}{\mathbf{X}}_{i}.$$

After the position-updating rule is implemented, the position (${{\mathbf{X}}^{^{\prime}}}_{i}$) is transformed from the eigen coordinate system back to the original coordinate system using Eq. (17)^53^.

17 $${\mathbf{X}}_{i+ \text{1} } = {\text{B}}{{\mathbf{X}}^{^{\prime}}}_{i}.$$

Lastly, Bujok^53^ proposed the archiving of good old positions for use in subsequent generations to prevent getting sticking around the local optima and to improve the exploration capacity. The idea of the archive is very simple. Archive A is introduced as empty, and in every selection step, when the current and new jellyfish positions are compared, the old (current) solution is added to A if the new position is better $\left(f\left({\mathrm{x}}_{i+ \text{1} }\text{)>}f( \, {\text{x}}_{i}\right)\right)$. When an archive of size N has been filled, the newly added old good solution randomly replaces one previously added. Notably, the storage of positions in A is assumed to occur in all three phases of JSO. Positions in A is used only in the following ocean current phase (Eq. (4)) to compute the average coordinates of the jellyfish population.

In the newly proposed JSO variant, the average vector is computed from the union of population *N* and archive A. Doing so reduces the speed of convergence (older good positions are used) but increases the probability of avoiding a local solution area, as formulated in Eq. (18)^53^.

18 $${\mathbf{X}}_{i+ \text{1} } = {\text{X}}_{i }+ rand\times \left({\mathbf{X}}_{\text{best}} \text{- }{\upbeta }{ \times }rand\times \frac{{\sum }_{i= \text{1} }^{N}{\mathbf{X}}_{i}}{N}\right),$$

where ${\mathbf{X}}_{i+ \text{1} }$ is the new position of the jellyfish; ${\mathbf{X}}_{i}$ is the current position of the jellyfish;${\mathbf{X}}_{\text{best}}$ is the position of the current best jellyfish in the swarm; *N* is swarm size, and $\beta$ is the distribution coefficient ($\beta$ > 0, $\beta$ = 3).

#### Exploitation boosting

The JSO search process is effective for exploration because the search pattern is sufficiently random for it. However, it sometimes works poorly for exploitation and so provides poor convergence. The search equation of the original JSO is based on a random selection of a solution vector from the current population that is combined randomly with the current solution vector in order to generate a new candidate solution vector. However, this search strategy is inefficient because one solution vector may contain good information on some dimensions while the other one may contain good information on other dimensions. Hence, some new JSO variants have been proposed.

Manita and Zermani^54^ proposed a modified version of the jellyfish search optimizer that is known as the orthogonal learning jellyfish search optimizer (OLJSO) to solve global optimization problems. The purpose of the proposed algorithm is to increase the exploitation ability of the JSO algorithm. The original JSO can be updated as OLJSO with an orthogonal learning strategy. This strategy is based on the orthogonal learning design (OLD) concept and addresses particularly the exploitation capability. The orthogonal learning design (OLD) finds the best candidate solution by testing only a few representative combinations from the search space.

The main idea behind this method of OLD is to use the properties of the fractional experiment to determine the best sequence of levels. Based on the orthogonal design, (M + 1) offspring (solutions) are generated and these are regarded as the new search mechanism. The purpose of this procedure is to improve the exploitation capacity of the original JSO algorithm. This new strategy begins with two candidate solutions. The first one is randomly selected from the current population. The other one will be generated as given by Eq. (19)^54^.

19 $${\mathbf{X}}_{i}\text{ = }{\textbf{X}}_{a}\text{ + }rand\left(\text{0,1}\right)\times \left({\mathbf{X}}_{b} -{\textbf{ X}}_{c}\right),$$

where,${\mathbf{X}}_{i}$ is the new location of a jellyfish; ${\mathbf{X}}_{a}$, ${\mathbf{X}}_{b}$ and ${\mathbf{X}}_{c}$ are randomly chosen locations that are determined by the population size (NP), $i\in${1, 2,…, NP}, *a*, *b*, *c* $\in$ {1, 2,…, NP}. The experimental results reveal that the proposed algorithm strongly outperforms the original algorithm in all respects, except for execution time.

Abdel-Basset et al.^55^ presented an improved version of JSO (IJSO) by integrating a novel method called the premature convergence strategy (PCS), to promote exploitation in search space and address the parameter extraction problem of photovoltaic (PV) models that are based on a single diode model (SDM) and a double diode model (DDM). This method works by preserving diversity among the members of the population while accelerating convergence toward the best solution based on two motions of the particles, which are as follows: (i) moving the current solution between two particles that are selected randomly from the population, and (ii) identifying the better of the best-so-far solution and a random solution from the population. These two motions are managed using a weight variable (a control variable) that is generated randomly in the range (0, 1). When the weight variable has a large value, the relative motion of two selected randomly particles is large, but if it is small, then the premature convergence method attends to the area between the best-so-far and the randomly selected solution.

The proposed PCS method improves the ability of the algorithm to exploit the best-so-far solution when the weight variable r is small and improves exploration around the particles to reach other regions when r is high. Mathematically, the premature convergence method is formulated as Eq. (20)^55^:

20 $${\mathbf{X}}_{i}\left({\text{t}}+ \text{1} \right)\text{ = }{\textbf{X}}_{i}\left(t\right)\text{ + r}\times \left({\mathbf{X}}_{{r}_{1}}(t)\text{ - }{\textbf{X}}_{{r}_{2}}(t)\right)+\left(\text{1 - r}\right)\times \left({\mathbf{X}}^{*} -{\textbf{ X}}_{{r}_{3}}(t)\right),$$

where *r*_1_, *r*_2_, and *r*_3_ are the indices of three solutions that are picked randomly from the population, and r is the control parameter and is a random number between (0, 1) that is used to control the motion of the current solution. If it is small, then the current solution moves to a location between the best-so-far and ${\mathbf{X}}_{{r}_{3}}(t)$ to accelerate convergence; if it is high, then the current solution is updated based on two randomly selected solutions from the population to improve the ability of the algorithm to reach other regions. This method is then combined with JSO to modify its performance to find better solutions in fewer iterations than the original JSO. Numerical simulations and results confirm the dominance of the proposed algorithm in terms of the accuracy and the rate of convergence.

Kaveh et al.^52^ proposed QJSO, to enhance the exploitation capability of the original JSO and accelerate its convergence without loss of diversity. The jellyfish exhibit passive (type A) motion around the location of the best jellyfish found so far, as follows^52^.

21 $${\mathbf{X}}_{i}(t)\text{ = }{\textbf{X}}^{\text{best}}+(\text{-1}{)}^{randi\left(\left[\text{1,2}\right]\right)}\times rand\left(\text{0,1}\right)\times \left({\mathbf{U}}_{{\varvec{b}}}-{\textbf{L}}_{{\varvec{b}}}\right),$$

where ${\mathbf{X}}_{i}(t)$ is the current location of the jellyfish;${\mathbf{X}}^{\text{best}}$ is the location of the best jellyfish found so far; and *randi* [1,2] returns a pseudorandom scalar integer in (1, 2). The term *randi* ([1,2]) allows exploration of the whole neighborhood of the best jellyfish found so far. Numerical results confirm that the proposed QJSO considerably outperforms the original JSO and has superior or comparable performance to those of other state-of-the-art optimization algorithms.

#### Simplified and enhanced time control mechanisms

Following ocean currents encourages diversification in the search space (global exploration), while exploitation of the search involves passive and active motions inside jellyfish swarms. Thus, the classical JSO seems to suffer from a lack of global exploration of the search space, focusing mainly on local exploitation of the best solutions found. In the quantum jellyfish search optimizer (QJSO), proposed by Kaveh et al.^52^, a better balance between diversification and intensification of the search process is achieved using a proposed simple linear time control mechanism as follows^52^.

22 $$\mathrm{c}\left(t\right)=\left|\left(\text{1 - }\frac{t}{\text{Maxiter}}\right)\right|.$$

Such a deterministic time control mechanism results in a simple trade-off between intensification and diversification during the search.

Truong and Chou^47^ proposed a fuzzy adaptive time control mechanism to improve exploration and exploitation during the movement of the jellyfish, where the ocean current rate (OCR) and the jellyfish swarm rate (JSR) are inputs and the output is bar movement bar(*t*). The bar(*t*) is used to tune the time control function c(*t*), according to the following equation^47^.

23 $$\text{c}\left(t\right)=\text{c}(\textit{t}-1)+\text{bar}(\textit{t} - 1),$$

where c(*t *− 1) is a time control function at time (*t* − 1) and c(0) = 0.5.

#### Handling of boundary conditions

In the classical JSO, if a solution exceeds the boundary of the search space, then the boundary handling mechanism of Eq. (10) brings it back to the opposite bound. Such a boundary handling mechanism may cause difficulties in the convergence process. Indeed, optimal solutions often lie close to (or even on) the boundary of the search space^39^. Thus, during the optimization process, especially in its final stages, many solutions, which are probably close to optimal, are likely to move beyond the boundaries of the search space. However, the boundary handling mechanism of Eq. (10) significantly alters the values of the design variables fall outside their corresponding bounds.

As a consequence of this boundary handling mechanism, some potentially good solutions, which fall slightly beyond the boundary of the search space, may be lost during the search process. QJSO, proposed by Kaveh et al.^52^, addresses this issue. The boundary handling mechanism of the original JSO that brings solutions back to the violated bound when they fall outside the boundary of the search space is updated using Eq. (24)^52^:

24 $${\mathbf{X}}_{i,\text{d}}^{{\prime}}= \left\{\begin{array}{c}{\mathbf{U}}_{\text{b,d}} \quad {\text{if}} \; {\textbf{X}}_{i,\text{d}} > {\textbf{U}}_{\text{b,d}}\\ {\mathbf{L}}_{\text{b,d}} \quad {\text{if}} \; {\textbf{X}}_{i,\text{d}} < {\textbf{L}}_{\text{b,d},}\end{array}\right.$$

where ${\mathbf{X}}_{i\text{,d}}^{^{\prime}}$ is the updated position of the jellyfish *i* with d dimensions of decision variables, and ${\mathbf{U}}_{\text{b, d}}$ and ${\mathbf{L}}_{\text{b, d}}$ are the upper and lower bounds, respectively.

### Hybridizing JSO with artificial intelligence

Shaheen et al.^57^ proposed the optimization of combined heat and power economic dispatch (CHPED) using a novel amalgamated heap-based and jellyfish optimizer (AHJFO). They improved the efficiency of two newly developed techniques, the heap-based optimizer (HBO) and JSO. Their proposed AHJFO incorporates an adjustment strategy function (ASF) to improve exploration in a few iterations by improving solutions that are generated using HBO. Further, as the iterations proceed, exploitation is improved by updating the solutions that are generated using JSO. The proposed AHJFO is more effective than HBO and JSO in solving the CHPED problem for medium-sized 24 unit and large 96-unit systems. Simulation results reveal the superiority of the proposed AHJFO over HBO, JSO, and other algorithms in solving CHPED problems.

Ginidi et al.^58^ proposed a heap-based jellyfish search optimizer (HBJSO) to enhance the performance of two recently developed algorithms: the heap-based optimizer (HBO) and JSO. The proposed hybrid HBJSO seeks to make use of the explorative features of HBO and the exploitative features of JSO to overcome some of the issues associated with their standard forms. The proposed hybrid HBJSO, HBO, and JSO were validated and statistically compared by using them to solve a real-world optimization problem of combined heat and power economic dispatch in electrical grids. The proposed hybrid HBJSO, HBO, and JSO were applied to two medium-sized systems of 24 and 48 units, and two large systems of 84- and 96-units. The experimental results demonstrated that the proposed hybrid HBJSO outperforms the standard HBO and JSO and other reported techniques in power economic dispatch.

Rajpurohit and Sharma^59^ proposed a hybrid metaheuristic (jellyfish search optimizer (JSO) and sine–cosine algorithm (SCA)) algorithm that combines the advantages of both constituents algorithms and outperforms them. They first used opposition-based learning in population initialization and then introduced a modified position update operator of the SCA into the original structure of JSO. They tested their proposed hybrid metaheuristic (JSO-SCA) on a set of benchmark functions to find the minimum weight design of a transmission tower, and it outperformed both JSO and SCA.

Utama et al.^60^ proposed the hybrid-jellyfish (HJF) algorithm which combines JSO and the neighborhood exchange strategy. HJF has five major stages: (1) initializing the position of the jellyfish swarm and ocean currents; (2) updating position based on jellyfish movement; (3) applying the time control mechanism; (4) applying the rules of large rank value; and (5) improving solutions based on neighborhood exchange procedures. Their proposed algorithm was used to solve the fuel consumption capacity vehicle routing problem (FCCVRP) and numerical results indicate that it effectively reduces the total cost of fuel.

Lei et al.^61^ proposed an enhanced algorithm, known as the fractional-order modified strategy and Gaussian mutation mechanism jellyfish search optimizer (FOGJSO). The fractional-order modified strategy and Gaussian mutation mechanism are integrated with the original JSO to electively enhance the convergence precision tracking performance of the original JSO. The proposed algorithm outperforms various classical methods on IEEE Congress on Evolutionary Computation (CEC) 2017 and CEC 2019 test functions with respect to accuracy, stability, and convergence acceleration.

### Multi‑objective version of JSO

Chou and Truong^46^ developed the multi-objective jellyfish search (MOJS) optimizer to solve engineering problems. MOJS integrates Lévy flight, an elite population, a fixed-size archive, a chaotic map, and the opposition-based jumping method to obtain Pareto-optimal solutions. These techniques are employed to define the motions of jellyfish in an ocean current or a swarm in multi-objective search spaces. They tested their proposed algorithm on 20 multi-objective mathematical benchmark problems and compared it with six well-known metaheuristic optimization algorithms such as MOALO (multi-objective antlion optimization), MOGWO (multi-objective grey wolf optimization), and MOPSO (multi-objective particle swarm optimization). They used MOJS to solve three constrained structural problems (25, 160, and 942-bar tower design) to minimize structural weight and maximum allowable nodal deflection. The results thus obtained indicate that the MOJS algorithm finds highly accurate approximations to Pareto-optimal fronts with a uniform distribution of solutions for the test functions. The advantages of using MOJS in solving real engineering problems and finding the best Pareto-optimal fronts are thus demonstrated.

Shaheen et al.^62^ proposed an enhanced multi-objective quasi-reflected JSO (MOQRJSO) for solving multi-dimensional optimal power flow (MDOPF) problems. They made the following two modifications to the standard JSO algorithm. (1) A cluster of a random size that represents the social community was proposed. Data are shared in the cluster and (2) quasi-opposition-based learning is incorporated into JSO to support the exploration phase. A fuzzy decision-making strategy is integrated into MOQRJSO to select the best solutions. The concept of Pareto optimality is used to extract the non-dominated solutions. The superiority of the MOQRJSO was proved by its application to an IEEE 30-bus system, an IEEE 57-bus system, the West Delta Region System of 52 buses (WDRS-52) in Egypt, and a large 118-bus system. The outcomes achieved using the proposed MOQRJSO were compared with those achieved using the conventional MOJS algorithm and other techniques in the literature. MOQRJSO yields lower fuel costs and power losses than those other techniques revealing its robustness, effectiveness, and superiority in handling MDOPF problems.

## Applications

This section focuses on the use of jellyfish search optimizer (JSO) in engineering optimization, prediction and classification, and the algorithmic fine-tuning of artificial intelligence.

### Engineering optimization

The jellyfish search optimizer (JSO) has been used in various engineering fields, such as power systems and energy generation, communication and networking, and civil and construction engineering as detailed below.

#### Power system and energy generation

This section examines the uses of JSO in power systems and energy-related fields. Rai and Verma^63^ used JSO to find the economic load dispatch of generating units, considering transmission losses. The effectiveness of the proposed method was evaluated by testing it on six systems under various loads. It was compared with the lambda-iterative and PSO algorithms to find the most efficient among them. JSO yielded the lowest fuel cost and transmission losses of the compared methods.

Tiwari et al.^64^ used JSO to analyze the effect of the installation of distributed generation (DG) and a capacitor bank (CB) on a radial distribution system (RDS). They carried out a cost-based analysis that considered the major expenses that are incurred due to the installation, operation, and maintenance of DG and CB units. They tested JSO on the IEEE 33-bus RDS. The results of their simulation were compared with those of simulations of other methods in the literature. JSO outperformed these other methods in terms of both power loss minimization and profit maximization.

Farhat et al.^65^ used JSO to propose a power flow model that included three types of energy sources, which were thermal power generators representing conventional energy sources, wind power generators (WPGs), and solar photovoltaic generators (SPGs). They used a modified IEEE 30-bus test system to determine its feasibility. To examine the effectiveness of the proposed power flow model, the results of its simulation were compared with the results of simulations of four other nature-inspired global optimization algorithms. The results established the effectiveness of JSO in solving the optimal power flow (OPF) problem with respect to both minimization of total generation cost and solution convergence.

Shaheen et al.^66^ introduced an efficient and robust technique that used JSO for optimal Volt/VAr coordination based on a joint distribution system reconfiguration (DSR)with the integration of distributed generation units (DGs) and the operation of distribution static VAr compensators (SVCs). JSO yielded the best solution and a comparison of the proposed JSO with similar approaches demonstrated its usefulness in modern control centers.

Alam et al.^67^ used JSO to track the global maximum power point (GMPP) of the solar photovoltaic (PV) module under partial shading conditions. Their results suggested that the JSO method has good tracking speed and accuracy. They also found that the JSO strategy tracks the GMPP in half of the time that is taken by the PSO algorithm under both uniform and shaded conditions.

Boutasseta et al.^68^ used the JSO technique to modify the voltage of a photovoltaic array using a boost direct current to direct current (DC–DC) converter. Their experimental results revealed that JSO performs well under both normal and disturbed operating conditions.

Abdulnasser et al.^69^ used JSO for the optimal sizing and placement of DGs and capacitor banks (CBs). To elucidate the efficiency of the proposed algorithm, they considered various cases; the allocation of CBs only, the allocation of DGs only, and the allocation of both CBs and DGs. The results thus obtained reveal that the JSO delivered the best results with respect to technical, economic, and emission objectives.

Ngo^70^ used JSO to solve the economic dispatch problem and to reduce costs and fuel consumption in power systems. To verify the feasibility and the effectiveness of the proposed scheme, they conducted two case studies to test the optimization performance of the proposed method from multiple economic perspectives. The validation results reveal that the proposed scheme provided more speed, convergence, and robustness than the methods to which it was compared.

Nusair et al.^71^ used JSO and other recently developed algorithms (slime mould algorithm (SMA), artificial ecosystem-based optimization (AEO), and marine predators algorithm (MPA)) to solve both multi- and single-OPF objective problems for a power network that incorporate flexible alternating current transmission system (FACTS) and stochastic renewable energy sources. They compared these algorithms to commonly available alternatives in the literature such as PSO, moth flame optimization (MFO), and grey wolf optimization (GWO), using an IEEE 30-bus test system. Their results reveal that JSO and other recently presented algorithms (MPA, SMA, and AEO) are more effective than PSO, GWO, and MFO in solving OPF problems.

Eid^72^ used JSO to allocate distributed generators (DG) and shunt capacitor (SC) banks optimally in distribution systems. He found JSO to be practical and effective in solving such nonlinear optimization problems, yielding better results than other algorithms in the literature. Huang and Lin^73^ used an improved jellyfish search optimizer (IJSO) to track the maximum power point (MPPT) under partial shade conditions. Their results showed that IJSO can accurately track the global maximum power point, and that it converged more quickly than an improved particle swarm optimization (IPSO) algorithm.

Shaheen et al.^57^ integrated a novel amalgamated heap-based agent with jellyfish optimizer (AHJFO) to optimize combined heat and power economic dispatch (CHPED). They improved the efficiency of two newly developed techniques: the heap-based optimizer (HBO) and JSO. AHJFO incorporates an adjustment strategy function (ASF) to improve exploration in a few iterations by improving the solutions that are generated using HBO. As the iterations proceed, exploitation is improved by updating solutions that are generated using JSO. AHJFO is more effective than HBO and JSO in solving the CHPED problem for medium-sized 24-unit and large 96-unit systems. Simulation results reveal the superiority of the proposed AHJFO over HBO, JSO and other algorithms for solving the CHPED problems.

Ginidi et al.^58^ proposed an innovative hybrid heap-based JSO (HBJSO) to improve upon the performance of two recently developed algorithms: the heap-based optimizer (HBO) and JSO. HBJSO uses the explorative features of HBO and the exploitative features of JSO to overcome some of the weaknesses of these algorithms in their standard forms. HBJSO, HBO, and JSO were validated and statistically compared by using them to solve a real-world optimization problem of combined heat and power (CHP) economic dispatch. HBJSO, HBO, and JSO were applied to two medium-sized 24-unit and 48-unit systems, and two large 84 unit and 96-unit systems. The experimental results demonstrate that the proposed hybrid HBJSO outperforms the standard HBO, JSO and other reported techniques when, applied to CHP economic dispatch.

Shaheen et al.^74^ proposed an enhanced quasi-reflection jellyfish optimization (QRJFO) algorithm for solving the optimal power flow problem. Fuel costs, transmission losses and pollutant emissions were considered as multi-objective functions. The performance of the proposed QRJFO algorithm was evaluated on the IEEE 57-bus, the practical West Delta Region system and a large IEEE 118-bus. Simulation results demonstrate the quality of the solutions and resilience of QRJFO.

Boriratrit et al.^75^ used jellyfish search extreme learning machine (JS-ELM), the Harris hawk extreme learning machine (HH-ELM), and the flower pollination extreme learning machine (FP-ELM) to increase accuracy and reduce overfitting in electric energy demand forecasting. Their results show that the JS-ELM provided a better minimum root mean square error than the state-of-the-art forecasting models.

Ali et al.^76^ presented an effective optimal sizing technique for a hybrid micro-grid using JSO. Their proposed sizing approach considers uncertainty associated with hybrid renewable resources. They investigated several operating scenarios to evaluate the effectiveness of the proposed approach and compared it to various optimization techniques. Their results demonstrate the applicability of JSO to their problem of interest.

Rai and Verma^77^ used JSO to solve a combined economic emission problem for an isolated micro-grid. They conducted tests on this micro-grid system, comprising traditional generators and renewable energy sources in two scenarios. They compared the results with those obtained using available algorithms to prove that the JSO algorithm was more effective than the others.

Yuan et al.^78^ used the improved jellyfish search optimizer and support vector regression (IJSO-SVR) to solve the problems of grid connection and power dispatching that are caused by non stationary wind power output. IJSO exhibits good convergence ability, search stability, and optimum-seeking ability, and it is more effective than conventional methods in solving optimization problems. The IJSO-SVR model outperformed other models in the literature and presents a more economical and effective means of optimizing wind power generation to solve problems with its uncertainty and can be used in grid power generation planning and power system economic dispatch.

Chou et al.^79^ used JSO and convolutional neural networks (CNNs) to evaluate the power generation capacity of plant microbial fuel cells (PMFCs) on building rooftops. Their results demonstrate the superior performance of JSO-optimized deep CNNs in learning image features and their consequent suitability for constructing models for estimating power generation by PMFCs.

#### Communication and networking

This section investigates the use of JSO in the field of communication and networking. Selvakumar and Manivannan^80^ used JSO to overcome the shortcomings of defragmentation in networking, and to improve the quality of network services. The proposed combination of proactive/reactive defragmentation approach and JSO (PR-DF-JSO) outperformed state-of-the-art spectrum defragmentation algorithms in terms of spectrum utilization, network efficiency, and quality of service offered based on the results of experiments and standard quality metrics. Specifically, lower spectrum fragmentation complexity, a better bandwidth fragmentation ratio, and less overall connection blocking were achieved.

Durmus et al.^81^ used swarm-based metaheuristic algorithms JSO, PSO, artificial bee colony (ABC), and the mayfly algorithm (MA), to determine the optimal design of linear antenna arrays. They conducted extensive experiments on the design of 10-, 16-, 24- and 32-element linear arrays and determined the amplitude and the positions of the antennas. They performed each of their experiments 30 times owing to the randomness of swarm-based optimizers, and their statistical results revealed that the novel algorithms JSO and MA outperformed the well-known PSO and ABC methods.

Aravind and Maddikunta^82^ proposed a novel optimal route selection model for use with the internet of things (IoT) in the field of healthcare that was based on an optimized adaptive neuro-fuzzy inference system (ANFIS). They selected optimal routes for medical data using a new self-adaptive jellyfish search optimizer (SA-JSO) that was an enhanced version of the original JSO algorithm^39^. Their model outperformed others.

#### Civil and construction engineering

Structural optimization has become one of the most important and challenging branches of structural engineering, and it has consequently received considerable attention in the last few decades^83^. Chou and Truong^39^ developed JSO, motivated by the behavior of jellyfish in the ocean for use in civil and construction engineering. They used JSO to solve structural optimization problems, including 25, 52, and 582-bar tower designs. Their results showed that JSO not only performed best but also required the fewest evaluations of objective functions. Therefore, JSO is potentially an excellent metaheuristic algorithm for solving structural optimization problems.

Chou and Truong^46^ expanded the framework of the single-objective jellyfish search (SOJS) algorithm to a multi-objective jellyfish search optimizer (MOJS) for solving engineering problems with multiple objectives. MOJS integrates Lévy flight, an elite population, a fixed-size archive, a chaotic map, and the opposition-based jumping method to obtain Pareto-optimal solutions. Three constrained structural problems (25, 160, and 942-bar tower designs) of minimizing structural weight and maximum nodal deflection have been solved using the MOJS algorithm. MOJS is an effective and efficient algorithm for solving multi-objective optimization problems in civil and construction engineering.

Kaveh et al.^52^ proposed a quantum-based JSO, named Quantum JSO (QJSO), for solving structural optimization problems. QJSO is used to solve frequency-constrained large cyclic symmetric dome optimization problems. The results thus obtained reveal that QJSO outperforms the original JSO and has superior or comparable performance to that of other state-of-the-art optimization algorithms.

Rajpurohit and Sharma^56^ proposed an enhancement of JSO by the implementation of chaotic maps in population initialization. They applied their enhanced JSO to three classical constrained engineering design problems. Analysis of the results suggests that the sinusoidal map outperforms other chaotic maps in JSO and helps to find efficiently the minimum weight design of a transmission tower.

Ezzeldin et al.^84^ used JSO to develop optimal strategies for the sustainable management of saltwater intrusion into coastal aquifers based on the finite element method (FEM). They tested the effectiveness of JSO by applying it to a real aquifer system in Miami Beach to maximize its total economic benefit and total pumping rate. JSO has also been used in a case study of the El-Arish Rafah aquifer, Egypt, to maximize the total pumping rate. The results in both cases were compared to relevant results in the literature, revealing that the JSO is an effective and efficient management tool.

Chou et al.^85^ used JSO and convolutional neural networks (CNNs) to predict the compressive strength of ready-mixed concrete. Their analytical results reveal that computer vision-based CNNs outperform numerical data-based deep neural networks (DNNs). Thus, the bio-inspired optimization of computer vision-based convolutional neural networks has promise for predicting the compressive strength of ready-mixed concrete.

Chou et al.^86^ presented jellyfish search optimizer (JSO)-XGBoost and symbiotic organisms search (SOS)-XGBoost for forecasting the nominal shear capacity of reinforced concrete walls in buildings. Their proposed methods outperform the ACI provision equation and grid search optimization (GSO)-XGBoost in the literature. Thus, they can be used to improve building safety, simplify a cumbersome shear capacity calculation process, and reduce material costs. Their systematic approach also provides a general framework for quantifying the performance of various mechanical models and empirical formulas that are used in design standards.

Truong and Chou^47^ proposed a novel fuzzy adaptive jellyfish search optimizer (FAJSO) for use in the stacking system (SS) of machine learning. They integrated the JSO, the fuzzy adaptive (FA) logic controller, and stacking ensemble machine learning. Its application to construction productivity, the compressive strength of a masonry structure, the shear capacity of reinforced deep beams, the axial strength of steel tube confined concrete, and the resilient modulus of subgrade soils was investigated. Their results indicate that the FAJSO-SS outperformed other methods. Accordingly, their proposed fuzzy adaptive metaheuristic optimized stacking system is effective for providing engineering informatics in the planning and design phase.

### Prediction and classification

Prediction and classification are required in a variety of areas that involve time series and cross-sectional data^87, 88^. This section concerns articles in which JSO has been used alone or integrated with machine/deep learning algorithms for prediction and classification.

Almodfer et al.^89^ employed a random vector functional link (RVFL) network that was optimized by JSO, AEO, MRFO, and SCA to predict the performance of a solar thermo-electric air-conditioning system (STEACS). Their results revealed that the RVFL-JSO outperformed the other algorithms in predicting all responses of the STEACS with a correlation coefficient of 0.948–0.999. They recommended its use for modeling STEACS systems.

Chou et al.^90^ used JSO to optimize the convolutional neural network (CNN) hyper-parameters to ensure the accuracy and stability of CNN in predicting power consumption. Their analytical results provide insights into the formulation of energy policy for management units and can help power supply agencies to distribute regional power in a way that minimizes unnecessary energy loss.

Barshandeh et al.^91^ utilized JSO and the marine predator algorithm (MPA) to develop a learning-automata (LA)-based hybrid algorithm for benchmark function optimization and solving data clustering problem. They applied the proposed algorithm to ten datasets and compared it with competing algorithms using various metrics; the hybrid algorithm outperformed. Desuky et al.^92^ used JSO to classify imbalanced and balanced datasets. They performed experiments on 18 real imbalanced datasets, and the proposed method performed comparably with well-known and recently developed techniques.

Chou and Truong^88^ tested JSO and other parameter-less algorithms (TLBO, SOS) by using them in the hyperparameters finetuning of least squares support vector regression (LSSVR) to develop a novel forecasting system. The linear time-series has been optimized using nonlinear machine learning models to identify historical patterns of regional energy consumption. Analytical results confirm that the proposed system, JSO-LSSVR, can predict multi-step-ahead energy consumption time series more accurately than can the linear model.

Chou et al.^93^ developed a weighted-feature least squares support vector regression (WFLSSVR) model that is optimized by JSO to predict the peak friction angle (shear strength) of fiber-reinforced soil (FRS), which is a popular material for use in building geotechnical structures. Their results showed that JSO-WFLSSVR outperformed baseline, ensemble, and hybrid machine learning models, as well as empirical methods in the literature. The JSO-WFLSSVR model is also effective for selecting features and can help geotechnical engineers to estimate the shear strength of FRS.

Hoang et al.^94^ implemented a support vector machine classifier that was optimized using JSO for the automatic classification of the severity of concrete spalling. It partitions input data into two classes, shallow spalling and deep spalling. Experimental results, supported by the Wilcoxon signed-rank test, reveal that the newly developed method is highly effective for classifying the severity of concrete spalling with an accuracy rate of 93.33%, an F1 score of 0.93, and an area under the receiver operating characteristic curve of 0.97.

Siddiqui et al.^95^ used JSO to calculate the optimum switching angle in the modulation range to eliminate desired lower-order harmonics in a multilevel inverter (MLI) voltage control application. The total harmonic distortion (THD) values of five-, seven-, and nine-level were computed using JSO and compared with those obtained using the powerful differential evolution (DE) algorithm. The results thus obtained clearly demonstrated that the output of an MLI in JSO exhibits THD that is superior to that in the output of DE for low and medium values of the modulation index.

Çetinkaya and Duran^96^ used JSO and other recently developed optimization algorithms [marine predators’ algorithm (MPA), tunicate swarm algorithm (TSA), mayfly optimization algorithm (MOA), chimp optimization algorithm (COA), slime mould optimization algorithm (SMOA), archimedes optimization algorithm (AOA), and equilibrium optimizer algorithm (EOA)] to improve the precision of the clustering-based segmentation of vessels. Simulation results of these algorithms exhibited similar convergence rates and error performances. Statistical analyses demonstrated that the stability and robustness of each metaheuristic approach sufficed to separate vessel pixels from the background pixels of a retinal image.

Wang and Gao^97^ used the multi-objective jellyfish search optimizer (MOJS) to determine the weights of kernel functions. According to their experimental result concerning three American solar sites, the proposed system that integrates with MOJS provided a higher interval coverage rate and a narrower interval width than those of other systems.

Zhao^98^ used single-objective JSO to classify brain function in human brain function parcellation. Experimental results show that that the new method not only has a greater searching ability than other partitioning methods, but also can obtain better spatial structures and stronger functional consistency.

Lei et al.^61^ proposed an enhanced algorithm, known as the fractional-order modified strategy and Gaussian mutation mechanism jellyfish search optimizer (FOGJSO), to predict rural resident income. They used FOGJSO to optimize the order of a discrete fractional time-delayed grey model for forecasting rural resident income. The results reveal that FOGJSO performed much better with respect to precision and convergence speed than did other methods.

Shubham et al.^99^ used JSO for clustering between a dish type stirling solar generator, a micro hydro turbine, a diesel generator, a flywheel energy storage device, a super magnetic energy storage device and an electric vehicle in a renewable energy based microgrid to stabilize the frequency and tie line power in the system. They compared the performance of the JSO based dual stage controller with those of the black widow optimization algorithm, GA and the PSO-based controller, with respect to overshoot, undershoot, settling time and figure of demerit. JSO outperformed other optimization algorithms when used to tune dual stage (1+PI)TID controller involving a microgrid-based electric vehicle.

### Finetuning of artificial intelligence

Hyper-parameter optimization is essential to the development of efficient models in machine learning and deep learning algorithms, as well as for quality control in industrial production^100, 101^. JSO is an efficient and innovative algorithm that is used in hyper-parameter optimization.

Chou et al.^102^ used JSO to optimize the hyper-parameters of a deep learning model that is called residual network (ResNet) and is used to classify the deflection of reinforced concrete beams, based on observations made by computer vision. Their work supports an innovative method that engineers can use to measure the deflection of reinforced concrete beams. The results of their analysis revealed that the proposed ResNet model that was optimized by JSO was more accurate than conventional ResNet.

Dhevanandhini and Yamuna^103^ used JSO to find the optimal coefficients of a discrete wavelet transform (DWT) to improve efficient multiple-video watermarking. They analyzed the performance of the proposed method using various metrics and compared it with the DWT-based watermarking approach, which it outperformed.

Elkabbash et al.^104^ proposed a novel detection system that was based on optimizing the random vector functional link (RVFL) using JSO, following the dimensional reduction of Android application features. They used JSO to determine the optimal configurations of RVFL to improve classification performance. The optimized RVFL minimized the runtime of the models with the best performance metrics.

Gouda et al.^105^ employed JSO to solve the problem of evaluating the parameters of the polymer exchange membrane fuel cells (PEMFCs) model. The maximum percentage voltage-biased error was ± 1% in all test cases, indicating that JSO can solve this problem more effectively than other algorithms.

Youssef et al.^106^ used JSO to estimate the parameters of a single-phase power transformer from the current and voltage under any load. They consider difference between the estimated and actual values as the main objective function that must be minimized. Experimental results revealed that the parameters of the transformer equivalent circuit were accurately obtained, indicating that the algorithm can be used to estimate the parameters of a single-phase transformer.

Kızıloluk and Sert^107^ adopted JSO to optimize the hyper-parameters of the Alex Net CNN model for feature extraction in the Faster R-CNN-JSO model for the early detection of hurricanes from satellite images. The purpose was to alert people about upcoming disasters and thus minimize casualties and material losses. Their results demonstrated that hyper-parameter optimization increased the detection performance of the proposed approach by 10% over that of Alex Net without optimized hyper-parameters. The average precision of Hurricane-faster R-CNN-JS was 97.39%, which was remarkably higher than those of other approaches.

Bisht and Sikander^108^ used JSO to optimize the parameters of the solar photovoltaic (PV) model. They used JSO to optimize the parameters of a single-diode PV model using various performance measures, such as PV characteristics, power-voltage, and current-voltage curves, relative error (RE), root mean square error (RMSE), mean absolute error (MAE), and normalized mean absolute error (NMAE). Their proposed technique provided better results than other techniques, with a lower RE, RMSE, MAE, and NMAE; it also converged rapidly.

Azam et al.^109^ utilized JSO to dampen out low-frequency oscillations (LFOs) by tuning the critical parameters of conventional lead-lag type power system stabilizers. JSO is used to tune time-domain simulations of the angular frequency, rotor angle, and control signal. They tested this method on two separate multimachine networks that were exposed to a three-phase fault, and compared it with two well-known optimization algorithms, called PSO and the backtracking search algorithm (BSA). Their results show that JSO provided better damping power system ratio than did the other algorithms. Moreover, the JSO-based approach converged in fewer iterations.

Raja and Periasamy^110^ presented the block chain and JSO-based deep generative adversarial neural network (DGANN) method for the distributed routing scheme of a wireless sensor network (WSN). They used the block chain routing protocol to detect and store packets and to transfer them from the source to the destination efficiently to improve the security and efficiency of the DGANN method. They used JSO to optimize the weight parameters of the DGANN method. The simulation results demonstrate that in the routing of a WSN, DGANN with optimized parameters outperforms others methods, such as the multidimensional scaling-map, the trust-aware routing protocol through multiple attributes, and dynamic rate-aware classified key distributional secure routing algorithms.

Usharani et al.^111^ used JSO to optimize the hyperparameters of long short-term memory (LSTM) networks to enhance the error metrics of the approximate multiplier. They used their proposed pre-trained LSTM model to generate approximate design libraries for the different truncation levels as a function of area, delay, power and error metrics. Their experimental results on an 8-bit multiplier with an image processing application reveals that the proposed approximate computing multiplier achieved a superior area and power reduction with very good error rates.

Nyong-Bassey and Epemu^112^ used JSO and PSO to identify servomechanism parameters using a two-step approach, involving a first-order transfer function and iterative minimization of a fitness score that is derived from the root mean squared error between the experimental and simulated position responses of the servomechanism of an equivalent state-space model structure. The simulated angular position step responses of the servomechanism that runs the JSO and PSO algorithms showed very closely with each other, in terms of root mean squared error. Table 2 summarizes recent advances in the application of the jellyfish search optimizer.

**Table 2:** Applications of jellyfish search optimizer in various fields.

Area	Application	Algorithm	Author
Power system and energy generation	Solving economic load dispatch problem	JSO	Rai and Verma^63^
	Minimization of power loss and maximization of profit for DG and CB	JSO	Tiwari et al.^64^
	Minimization of total generation cost and solution convergence for OPF	JSO	Farhat et al.^65^
	Optimal Volt/VAr coordination, DG integration SVC operation	JSO	Shaheen et al.^66^
	Tracking GMPP of solar photovoltaic module	JSO	Alam et al.^67^
	Tracking optimal point of solar energy generation system	JSO	Boutasseta et al.^68^
	Optimizing both location and size of CB and DG	JSO	Abdulnasser et al.^69^
	Solving economic load dispatch problem	JSO	Ngo^70^
	Combined heat and power (CHP) economic dispatch	HBJSO	Ginidi et al.^58^
	Tracking MPPT under partial shade conditions	IJSO	Huang and Lin^73^
	Electrical energy demand forecasting	JSO	Boriratrit et al.^75^
	Optimal sizing technique for a hybrid micro-grid	JSO	Ali et al.^76^
	Addressing the problems of grid connection and power dispatching caused by non-stationary wind power output	IJSO-SVR	Yuan et al.^78^
	Evaluation of power generation capacity of plant microbial fuel cells (PMFCs)	JSO	Chou et al.^79^
	Solving combined economic emission problem for isolated micro-grid	JAO	Rai and Verma^77^
	Optimizing combined heat and power economic dispatch (CHPED)	AHJFO	Shaheen et al.^57^
	Solving optimal power flow (OPF) problem	QRJFO	Shaheen et al.^74^
	Determining minimum weight design of transmission tower	JSO-SCA	Rajpurohit and Sharma^59^
	Determining fuel consumption capacity of vehicles	HJSO	Utama et al.^60^
Communication and networking	Improving quality of service of the network	JSO	Selvakumar and Manivannan^80^
	Optimal design of linear antenna arrays	JSO & MA	Durmus et al.^81^
	Optimal route selection model in internet of things (IoT) for healthcare	SA-JSO	Aravind and Maddikunta^82^
Civil and construction engineering	Forecasting compressive strength of ready-mixed concrete	JSO	Chou et al.^79^
	Developing stacking system of machine learning for engineering planning and design	FAJSO	Truong and Chou^47^
	Solving structural design problems	JSO	Chou and Truong^39^
	Solving large cyclic symmetric dome optimization problems	QJSO	Kaveh et al.^52^
	Determining nominal shear capacity of reinforced concrete walls in buildings	JSO-XGBoost	Chou et al.^86^
	Solving structural optimization problems	MOJS	Chou and Truong^46^
	Solving classical constrained engineering design problems	JSO	Rajpurohit and Sharma^56^
Prediction and classification	Optimizing CNN hyper-parameters	JSO	Chou et al.^90^
	Predicting peak friction angle of fiber-reinforced soil (FRS)	JSO-WFLSSVR	Chou et al.^93^
	Classification of concrete as shallow or deep spalling	JSO	Hoang et al.^94^
	Clustering renewable energy based microgrid	JSO	Shubham et al.^99^
	Predicting optimal switching angle in voltage control	JSO	Siddiqui et al.^95^
	Predicting performance of STEACS	RVFL-JSO	Almodfer et al.^89^
	Benchmark function optimization and data clustering	LA-JSO	Barshandeh et al.^91^
	Classifying imbalanced and balanced datasets	JSO	Desuky et al.^92^
	Integrated interval forecasting for solar radiation	MOJS	Wang and Gao^97^
	Classifying human brain functions	JSO	Zhao^98^
	Forecasting income of rural residents	FOGJSO	Lei et al.^61^
Finetuning of artificial intelligence	Optimizing hyper-parameters of deep learning	JSO	Chou et al.^102^
	Optimizing hyper-parameters of LSSVR	JSO-LSSVR	Chou and Truong^88^
	Estimating parameters of a single-phase power transformer	JSO	Youssef et al.^106^
	Optimizing parameters of solar photovoltaic (PV) model	JSO	Bisht and Sikander^108^
	Finding optimal coefficients of DWT	JSO	Dhevanandhini and Yamuna^103^
	Finding optimal configurations of RVFL	JSO	Elkabbash et al.^104^
	Identification of parameters of PEMFCs	JSO	Gouda et al.^105^
	Optimizing hyper-parameters of Alex Net CNN model for extracting features in Faster R-CNN	JSO	Kızıloluk and Sert^107^
	Optimizing damped-out low-frequency oscillations (LFOs)	JSO	Azam et al.^109^
	Optimizing weight parameters in DGANN method	JSO	Raja and Periasamy^110^
	Identifying servomechanism parameters	JSO & PSO	Nyong-Bassey and Epemu^112^
	Optimizing hyperparameters of long short-term memory (LSTM) networks	JSO	Usharani et al.^111^

## Discussion

This section presents the findings of this study in relation to the research goals, including recent trends in the use of JSO, potential enhancements in JSO, and JSO variants, which are comparatively analyzed.

### Recent trends in use of JSO

**Figure 5:**
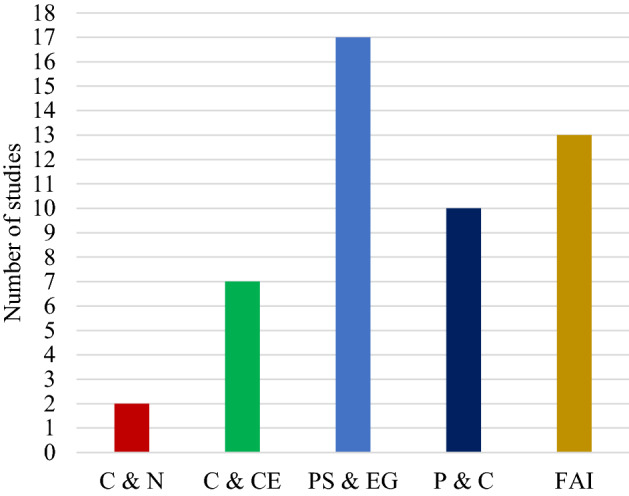
Number of studies related to applications of JSO.

This section compares recent research works on JSO and its variants in a variety of fields. JSO is a newly developed robust algorithm that has a good population utilization rate and maintains a favorable balance between exploration and exploitation. Optimization problems vary among fields and the No Free Lunch theorem specifies that a single algorithm cannot satisfactorily solve all such problems. The literature clearly reveals that JSO is being increasingly used since its source code was recently shared in the academic community. The use of JSO for optimization has attracted substantial interest since its initial development. However, the number of relevant works on MOJS was lower than that of the single objective JSO both with respect to theoretical frameworks and applications. Figure 5 presents the number of studies related to each area in which JSO is applied, where C & N = Communication and Networking; C & CE = Civil and Construction Engineering; PS & EG = Power System and Energy Generation; P & C = Prediction and Classification; and FAI = Finetuning of Artificial Intelligence.

According to Table 3, JSO and its variants are mostly applied to solve problems that are related to (1) engineering optimization (56%), including in power system and energy generation, communication and networking, and civil and construction engineering; (2) prediction and classification (21%); and (3) the finetuning of artificial intelligence (23%). Despite the favorable performance of JSO in solving many standard benchmark functions, CEC functions, and real-world constrained and unconstrained problems, the algorithm has been seldom applied to communication networks, portfolio optimization in finance, civil engineering infrastructure operations, and multiobjective optimization problems. Such applications may provide great opportunities in decision making and engineering management.

**Table 3:** Applications of JSO.

Area	Percentage (%)
Power system and energy generation	37
Communication and networking	6
Civil and construction engineering	13
Prediction and classification	21
Finetuning of artificial intelligence	23

JSO has been hybridized with other recently developed algorithms, such as the proactive and reactive defragmentation algorithm (PDRFA), the heap-based optimizer (HBO) and the convolutional neural network (CNN), to improve its performance and to optimize the hyper-parameters in machine and deep learning methods. However, JSO has not yet been hybridized with other optimization algorithms such as particle swarm optimization (PSO) or the artificial bee colony (ABC) algorithm, or with traditional techniques, such as dynamic programming, or other techniques, such as adaptive reinforcement learning.

### Potential enhancements of JSO

JSO has been shown to be efficient in solving numerous standard benchmark problems and real-world constrained and unconstrained applications. JSO has a good population utilization rate and maintains a favorable balance between global and local searches. However, the exploration/exploitation ability and convergence speed of the algorithm can be improved. Accordingly, researchers have developed numerous enhanced versions of JSO by upgrading its original mechanisms with artificial intelligent agents. Numerous variants or enhancements have been made by developing theoretical supplements to achieve diverse objectives.

The preceding sections of this study reported on these refinements, which have concerned the updating mechanisms in the diversity of population, the boosting agents of exploration and exploitation, improvements in the distribution coefficient for global search, the time control mechanism, and the handling of boundary conditions. The improvements include the orthogonal learning JSO (OLJSO) with an orthogonal learning strategy, the premature convergence strategy (PCS), archives of good old, solutions (positions), dynamic diversity control, and the quantum-based update rule.

### Comparative analysis of JSO variants

This section compares jellyfish search optimizer (JSO) with the different variants and hybrids of JSO that are applied in global optimization and real-world applications. The key features of each algorithm, the problems that are solved by it and its performance are considered. Table 4 exhibits a comparative analysis of variants of JSO. The JSO variants are comparable to, or better than, the classical JSO algorithm. Notably, the standard JSO shows better performance than some hybrid optimization algorithms, such as the proactive and reactive defragmentation-JSO algorithm (PRDA-JSO) and the spectrum defragmentation algorithm (SDA).

**Table 4:** Comparative analysis of variants and hybrids of JSO.

Author	Algorithm	Key features	Problems solved	Performance
Chou and Truong^46^	MOJS	Pareto-optimal solutions	Structural optimization problems	Performs better than MOALO, NSGA II, and many other multiobjective algorithms
Manita and Zermani^54^	OLJSO	Global searching of JSO	Evaluation of benchmark functions	OLJSO > JSO
Abdel-Basset et al.^55^	IJSO	Increased convergence speed	Optimization of solar/PV generating units	IJSO > JSO
Bujok^53^	3-Steps in JSO	Updates archive of good old solutions; controls population diversity	Solving real-world problems of CEC 2011	JSeigDiA, JSeig JSeigDi > GWO, JS, SOMA, ABC, PSO, TSA, FFL
Kaveh et al.^52^	QJSO	Applies quantum update rules to diversification, boundary, and time control mechanism	Structural optimization problems	QJSO > JSO
Ginidi et al.^58^	HBJSO	Updates explorative and exploitative features	Combined heat and power (CHP) economic dispatch	HBJSO > HBA, JSO, WOA
Shaheen et al.^62^	MOQRJSO	Enhances exploration phase on Pareto optimality	Multi-dimensional optimal power flow	MOQRJSO > JSO
Selvakumar and Manivannan^80^	PRDFJSO	Reduces probability of disruption and reconfiguration in network	Spectrum utilization, network efficiency, and quality of service	PRDFJSO > PRDFA, SDA
Shaheen et al.^57^	AHJFO	Improves the produced exploitation and its solutions	Combined heat and power economic dispatch (CHPED)	AHJFO > JSO, HO, GSA, PSO, GA
Rajpurohit and Sharma^59^	JSO-SCA	Population initialization and introduction of a modified position update operator	Minimum weight design of transmission tower	JSO-SCA > JSO and SCA
Utama et al.^60^	HJSO	Generates population parameter	Minimum cost of fuel consumption	HJSO > hybrid PSO > Hybrid TS > ELS
Rajpurohit and Sharma^56^	Chaotic JSO	Modifies active swarm motion	Benchmark evaluation	Chaotic JSO > JSO
Lei et al.^61^	FOGJSO	Fractional-order modified strategy and Gaussian mutation mechanism	Forecast income of rural residents	FOGJSO > JSO, PSO, DE, LSA, GBO, SOA, HGS, SSA, HBO, WHOA, and AOA
Truong and Chou^47^	FAJSO	Improves time control mechanism in JSO	Stacking machine learning for engineering planning and design	FAJS-SS_LSSVR_ > FAJS-SS_RBFNN_, PSO-SS_LSSVR_, TLBO-SS_LSSVR_

## Conclusion and recommendations

Research on JSO was reviewed. The underlying inspiration for JSO, its population initialization and boundary conditions, mathematical formulations of ocean currents and jellyfish swarms, time control mechanism, advantages and disadvantages, variants, and applications were thoroughly discussed. Analytical findings reveal that JSO is used in various disciplines, including power systems and energy generation, communication and networking, civil and construction engineering, prediction and classification, and fine-tuning of artificial intelligence.

Experimental research has established that JSO outperforms many nature-inspired algorithms, such as PSO, DE, AEO, MRFO, SCA, WOA, TLBO, ABC, and GA, in a variety of ways^39^. Some variants or enhancements have been made by considering the diversity of population, the updating mechanisms in exploration- or exploitation-oriented boosting, the population distribution, the time control mechanism, and the handling of boundary conditions.

Future work on JSO should consider the following: (1) Self-adaption: adaptive or self-adaptive algorithms are those that can self-tune their algorithm-specific and common control parameters. The algorithm-specific parameters in JSO include the number of iterations, population size, spatial distribution coefficients, and motion coefficients. Most relevant research has used trial-and-error experiments or sensitivity analyses to evaluate these algorithmic parameters, which are time-consuming. Accordingly, an adaptive version of JSO with the capacity to self-tune its algorithm-specific parameters is required. (2) Hybridization: in general, hybrid algorithms outperform stand-alone algorithms. Therefore, the hybridization of JSO with other conventional algorithms such as GA, PSO, ABE, DE, dynamic programming, and adaptive reinforcement learning, is a potential avenue for research. (3) Applications: JSO is currently utilized to solve a subset of complex optimization problems. More attempts to solve complicated optimization problems in the real world using JSO and its variants should be made to demonstrate the generalizability of JSO.

JSO yields competitive solutions to other complicated problems. Numerous ongoing studies have focused on minimizing the overall maintenance cost of buildings and roads, scheduling in civil and industrial engineering, and investment portfolio optimization problems in finance with limited constraints. JSO can be extended by hybridization in ways that depend on the problem to be solved. The findings in this review can therefore be used to promote future advances by considering the applications, advantages, and improvements of similar metaheuristic optimization algorithms thus developed. This work supports the theoretical framework of an enhanced version of JSO or other newly proposed optimizer that improves upon present and the original variants. It will also motivate researchers to develop novel bio-inspired metaheuristic optimization algorithms with tradeoff modifications.

## Data Availability

The data that support this study are available from the corresponding author upon reasonable request.

## Abbreviations

3-SJSO: Three-step in jellyfish search optimizer
ABC: Artificial bee colony
ANFIS: Adaptive neuro-fuzzy inference system
AEO: Artificial ecosystem optimizer
AHJFO: Amalgamated heap-based and jellyfish optimizer
AHOT: Artificial hummingbird optimization technique
AI: Artificial intelligence
ALOA: Antlion optimization algorithm
AOA: Archimedes optimization algorithm
ASF: Adjustment strategy function
AVOA: African vultures optimization algorithm
BSA: Backtracking search algorithm
CASM-JSO: Chaotic active swarm method-based jellyfish search optimizer
CB: Capacitor bank
CGO: Chaos game optimization
CHPED: Combined heat and power economic dispatch
CLMPA: Comprehensive learning marine predator algorithm
CNN: Convolutional neural network
COA: Chimp optimization algorithm
CSOAOA: Crisscross optimization and arithmetic optimization algorithm
DBOA: Dynamic butterfly optimization algorithm
DC-DC: Direct current to direct current
DDM: Double diode model
DE: Differential evolution
DG: Distributed generation
DGANN: Deep generative adversarial neural network
DGO: Dynamic group optimization
DMOA: Dwarf mongoose optimization algorithm
DNN: Deep neural network
DSR: Distribution system reconfiguration
DWT: Discrete wavelet transform
ELS: Evolutionary local search
EOA: Equilibrium optimizer algorithm
ESSA: Enhanced sparrow search algorithm
ESSOA: Enhanced shuffled shepherd optimization algorithm
FACTS: Flexible alternating current transmission system
FAJSO: Fuzzy adaptive jellyfish search optimizer
FBI: Forensic-based investigation algorithm
FCCVRP: Fuel consumption capacity vehicle routing problem
FEM: Finite element method
FMHHO: Fractional-order modified Harris hawk optimizer
FOGJSO: Fractional-order and Gaussian mutation mechanism jellyfish search optimizer
FPA: Flower pollination algorithm
FP-ELM: Flower pollination extreme learning machine
FRS: Fiber-reinforced soil
GA: Genetic algorithm
GBO: Gradient-based optimizer
GMPP: Global maximum power point
GPC: Giza pyramids construction
GSO: Grid search optimization
GTO: Gorilla troops optimizer
GTOA: Group teaching optimization algorithm
GWO: Grey wolf optimizer
HAFA: Heuristic-based approaches fragmentation aware
HB-JSO: Heap-based and jellyfish search optimizer
HBO: Heap-based optimizer
HGS: Hunger games search
HH-ELM: Harris hawk extreme learning machine
HJF: Hybrid-jellyfish
HMI: Harmonics multilevel inverter
HMPA: Hybrid marine predator algorithm
HPCSA: High-performance cuckoo search algorithm
HSSATLBO: Hybrid salp swarm algorithm with teaching–learning-based optimization
ICHOA: Improved chimp optimization algorithm
IJSO: Improved jellyfish search optimizer
IJSO-SVR: Improved jellyfish search optimizer and support vector regression
IoT: Internet of things
IPSO: Improved particle swarm optimization
JSO: Jellyfish search optimizer
JSO-XGBoost: Jellyfish search optimizer-XGBoost
LA: Learning-automata
LFO: Low-frequency oscillations
LSA: Lightning search algorithm
LSSVR: Least squares support vector regression
MDOPF: Multi-dimensional optimal power flow
MFO: Moth flame optimization
MJSO: Modified jellyfish search optimizer
MMRFOA: Modified manta ray foraging optimization algorithm
MOA: Mayfly optimization algorithm
MOALO: Multi-objective ant lion optimizer
MOGWO: Multi-objective grey wolf optimization
MOJS: Multi-objective jellyfish search
MOPSO: Multi-objective particle swarm optimization
MOQRJSO: Multi-objective quasi-reflected jellyfish search optimizer
MPA: Marine predators algorithm
MPPT: Maximum power point track
MRFO: Manta ray foraging optimization
OLD: Orthogonal learning design
OLJSO: Orthogonal learning jellyfish search optimizer
OPF: Optimal power flow
PCS: Premature convergence strategy
PEMFC: Polymer exchange membrane fuel cells
PMFC: Plant microbial fuel cell
PRDF: Proactive and reactive defragmentation
PRDFA: Proactive and reactive defragmentation algorithm
PSO: Particle swarm optimization
PSPSO: Partitioned step particle swarm optimization
PV: Photovoltaic
QJSO: Quantum jellyfish search optimizer
RDS: Radial distribution system
RVFL: Random vector functional link
SA-JSO: Self-adaptive jellyfish search optimizer
SAO: Smell agent optimization
SC: Shunt capacitor
SCA: Sine cosine algorithm
SDA: Spectrum defragmentation algorithm
SDM: Single diode model
SMA: Slime mould algorithm
SMO: Starling murmuration optimizer
SOA: Seagull optimization algorithm
SOJS: Single objective jellyfish search
SOS: Symbiotic organisms search
SOS-XGBoost: Symbiotic organisms search-XGBoost
SPVG: Solar photovoltaic generator
SS: Stacking system
SSA: Sparrow search algorithm
STEACS: Solar thermoelectric air-conditioning system
SVC: Static var compensator
THD: Total harmonic distortion
TLBO: Teaching-learning-based optimization
TLS-PSO: Three-learning strategy particle swarm optimization
TS: Tabu search
TSA: Tunicate swarm algorithm
WDRS: West delta region system
WFLSSVR: Weighted-feature least squares support vector regression
WHOA: Wild horse optimization algorithm
WOA: Whale optimization algorithm
WPG: Wind power generator
WSN: Wireless sensor network
WSOA: War strategy optimization algorithm
